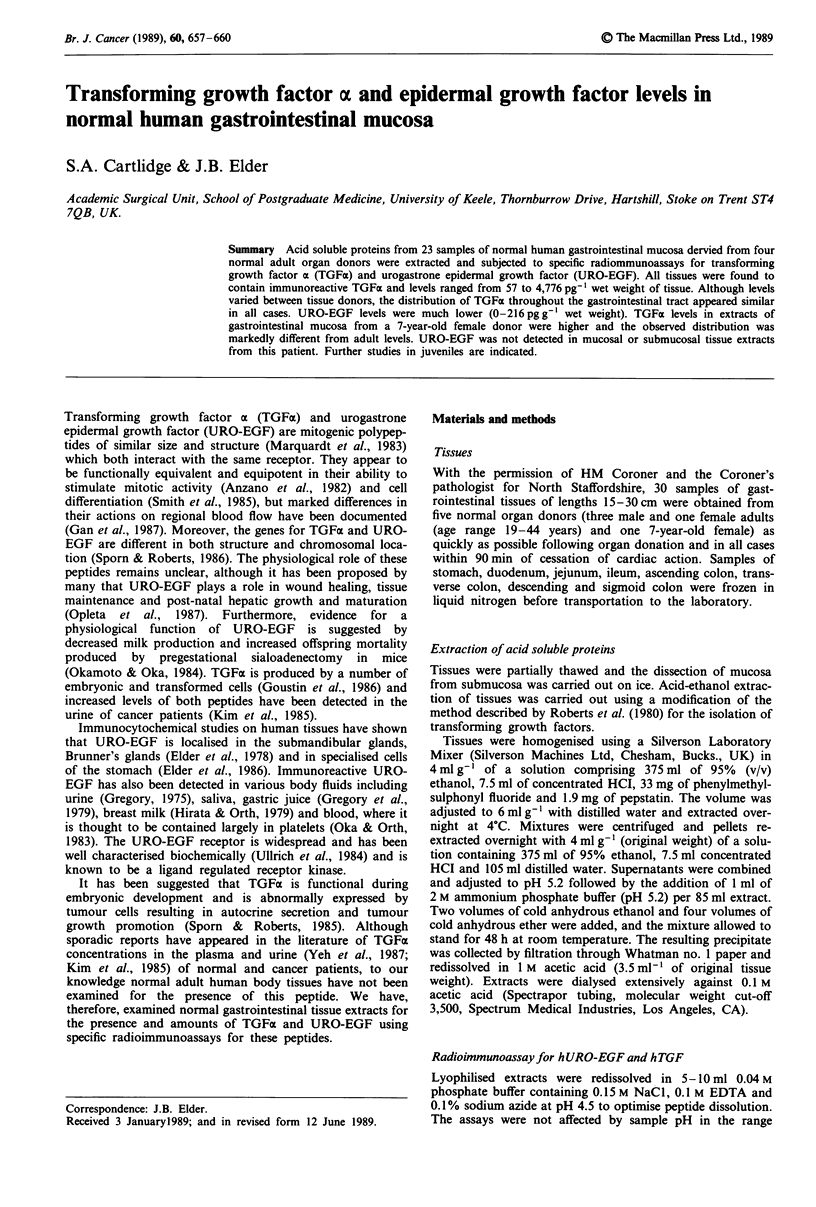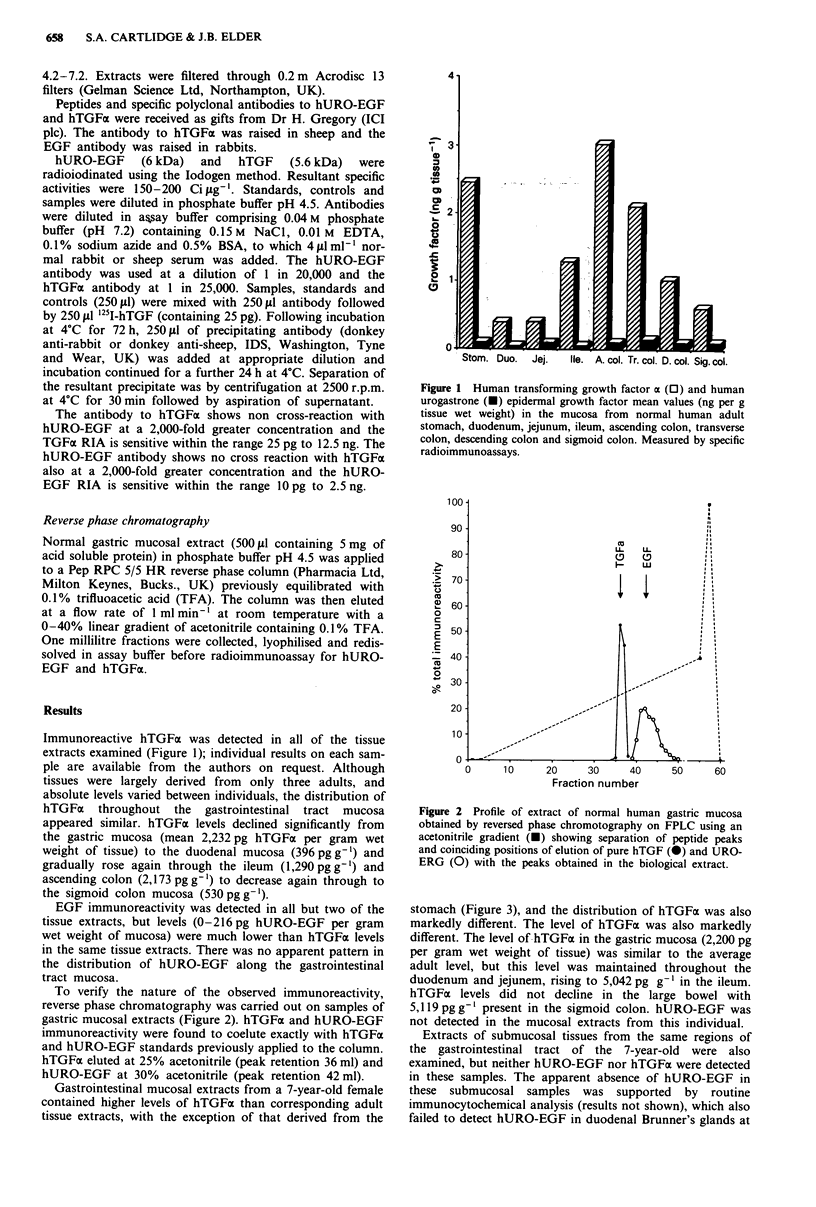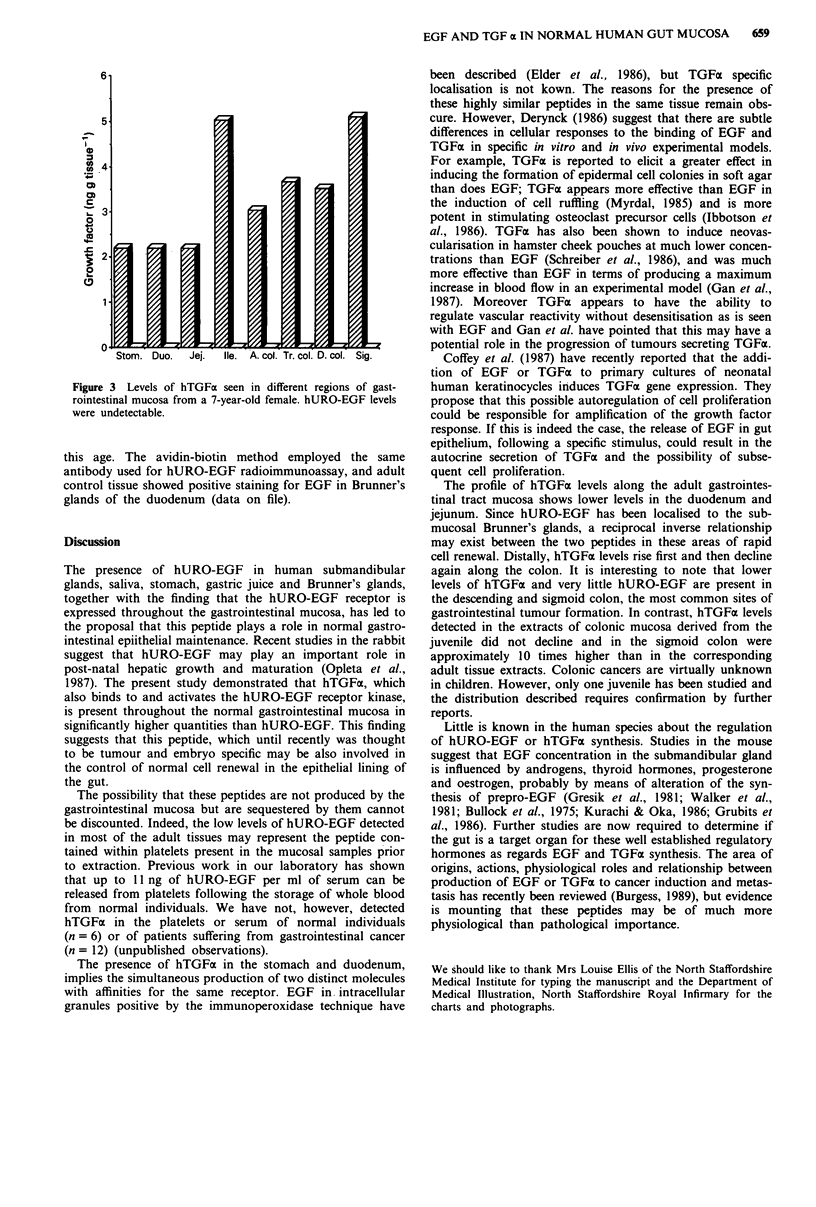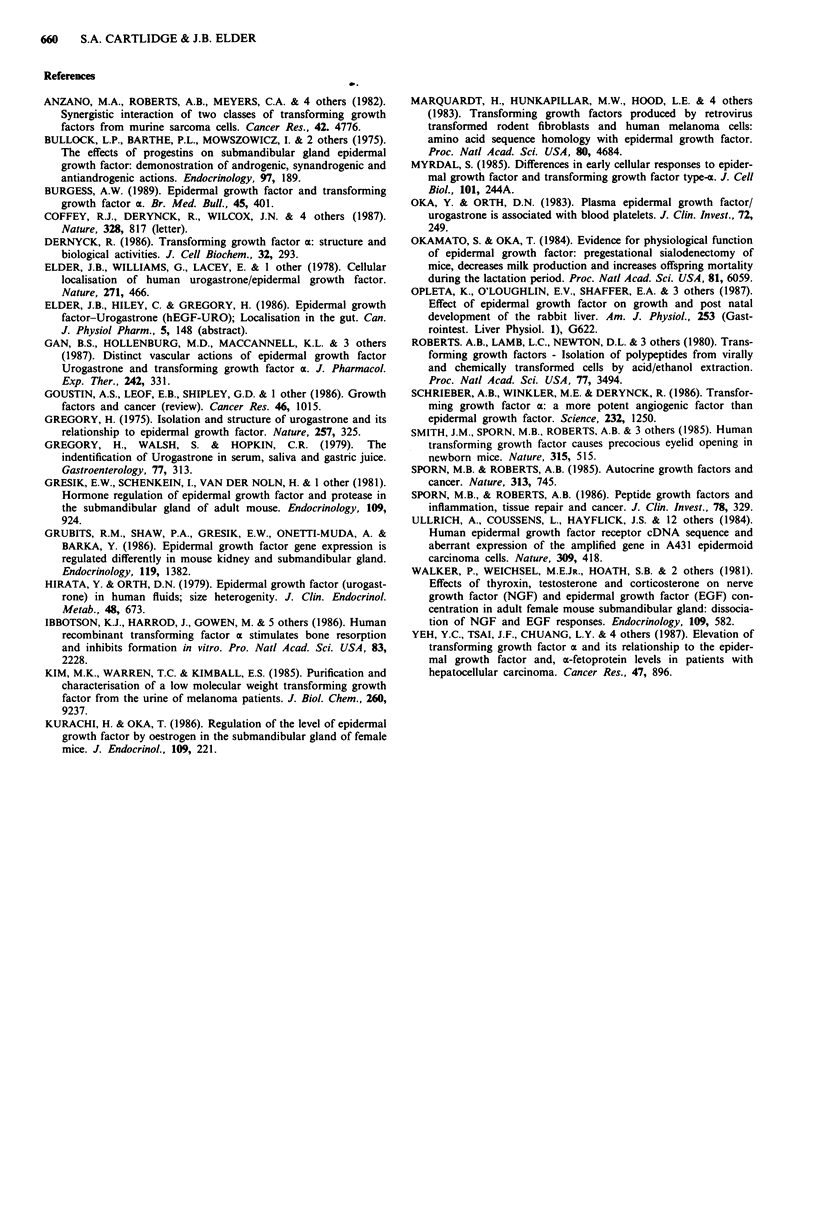# Transforming growth factor alpha and epidermal growth factor levels in normal human gastrointestinal mucosa.

**DOI:** 10.1038/bjc.1989.334

**Published:** 1989-11

**Authors:** S. A. Cartlidge, J. B. Elder

**Affiliations:** Academic Surgical Unit, School of Postgraduate Medicine, University of Keele, Hartshill, Stoke on Trent, UK.

## Abstract

Acid soluble proteins from 23 samples of normal human gastrointestinal mucosa derived from four normal adult organ donors were extracted and subjected to specific radiommunoassays for transforming growth factor alpha (TGF alpha) and urogastrone epidermal growth factor (URO-EGF). All tissues were found to contain immunoreactive TGF alpha and levels ranged from 57 to 4,776 pg-1 wet weight of tissue. Although levels varied between tissue donors, the distribution of TGF alpha throughout the gastrointestinal tract appeared similar in all cases. URO-EGF levels were much lower (0-216 pg g-1 wet weight). TGF alpha levels in extracts of gastrointestinal mucosa from a 7-year-old female donor were higher and the observed distribution was markedly different from adult levels. URO-EGF was not detected in mucosal or submucosal tissue extracts from this patient. Further studies in juveniles are indicated.


*Br. J. Cancer* (1989), **60**, 657–660 © The Macmillan Press Ltd., 1989

### Transforming growth factor α and epidermal growth factor levels in normal human gastrointestinal mucosa

S.A. Cartlidge & J.B. Elder

*Academic Surgical Unit, School of Postgraduate Medicine, University of Keele, Thornburrow Drive, Hartshill, Stoke on Trent ST4 7QB, UK.*

**Summary** Acid soluble proteins from 23 samples of normal human gastrointestinal mucosa dervied from four normal adult organ donors were extracted and subjected to specific radiommunoassays for transforming growth factor α (TGFα) and urogastrone epidermal growth factor (URO-EGF). All tissues were found to contain immunoreactive TGFα and levels ranged from 57 to 4,776 pg$^{-1}$ wet weight of tissue. Although levels varied between tissue donors, the distribution of TGFα throughout the gastrointestinal tract appeared similar in all cases. URO-EGF levels were much lower (0–216 pg g$^{-1}$ wet weight). TGFα levels in extracts of gastrointestinal mucosa from a 7-year-old female donor were higher and the observed distribution was markedly different from adult levels. URO-EGF was not detected in mucosal or submucosal tissue extracts from this patient. Further studies in juveniles are indicated.

Transforming growth factor α (TGFα) and urogastrone epidermal growth factor (URO-EGF) are mitogenic polypeptides of similar size and structure (Marquardt *et al.*, 1983) which both interact with the same receptor. They appear to be functionally equivalent and equipotent in their ability to stimulate mitotic activity (Anzano *et al.*, 1982) and cell differentiation (Smith *et al.*, 1985), but marked differences in their actions on regional blood flow have been documented (Gan *et al.*, 1987). Moreover, the genes for TGFα and URO-EGF are different in both structure and chromosomal location (Sporn & Roberts, 1986). The physiological role of these peptides remains unclear, although it has been proposed by many that URO-EGF plays a role in wound healing, tissue maintenance and post-natal hepatic growth and maturation (Opleta *et al.*, 1987). Furthermore, evidence for a physiological function of URO-EGF is suggested by decreased milk production and increased offspring mortality produced by pregestational sialoadenectomy in mice (Okamoto & Oka, 1984). TGFα is produced by a number of embryonic and transformed cells (Goustin *et al.*, 1986) and increased levels of both peptides have been detected in the urine of cancer patients (Kim *et al.*, 1985).

Immunocytochemical studies on human tissues have shown that URO-EGF is localised in the submandibular glands, Brunner's glands (Elder *et al.*, 1978) and in specialised cells of the stomach (Elder *et al.*, 1986). Immunoreactive URO-EGF has also been detected in various body fluids including urine (Gregory, 1975), saliva, gastric juice (Gregory *et al.*, 1979), breast milk (Hirata & Orth, 1979) and blood, where it is thought to be contained largely in platelets (Oka & Orth, 1983). The URO-EGF receptor is widespread and has been well characterised biochemically (Ullrich *et al.*, 1984) and is known to be a ligand regulated receptor kinase.

It has been suggested that TGFα is functional during embryonic development and is abnormally expressed by tumour cells resulting in autocrine secretion and tumour growth promotion (Sporn & Roberts, 1985). Although sporadic reports have appeared in the literature of TGFα concentrations in the plasma and urine (Yeh *et al.*, 1987; Kim *et al.*, 1985) of normal and cancer patients, to our knowledge normal adult human body tissues have not been examined for the presence of this peptide. We have, therefore, examined normal gastrointestinal tissue extracts for the presence and amounts of TGFα and URO-EGF using specific radioimmunoassays for these peptides.

Correspondence: J.B. Elder.
Received 3 January1989; and in revised form 12 June 1989.

### Materials and methods

### *Tissues*

With the permission of HM Coroner and the Coroner's pathologist for North Staffordshire, 30 samples of gastrointestinal tissues of lengths 15–30 cm were obtained from five normal organ donors (three male and one female adults (age range 19–44 years) and one 7-year-old female) as quickly as possible following organ donation and in all cases within 90 min of cessation of cardiac action. Samples of stomach, duodenum, jejunum, ileum, ascending colon, transverse colon, descending and sigmoid colon were frozen in liquid nitrogen before transportation to the laboratory.

### *Extraction of acid soluble proteins*

Tissues were partially thawed and the dissection of mucosa from submucosa was carried out on ice. Acid-ethanol extraction of tissues was carried out using a modification of the method described by Roberts *et al.* (1980) for the isolation of transforming growth factors.

Tissues were homogenised using a Silverson Laboratory Mixer (Silverson Machines Ltd, Chesham, Bucks., UK) in 4 ml g$^{-1}$ of a solution comprising 375 ml of 95% (v/v) ethanol, 7.5 ml of concentrated HCl, 33 mg of phenylmethylsulphonyl fluoride and 1.9 mg of pepstatin. The volume was adjusted to 6 ml g$^{-1}$ with distilled water and extracted overnight at 4°C. Mixtures were centrifuged and pellets re-extracted overnight with 4 ml g$^{-1}$ (original weight) of a solution containing 375 ml of 95% ethanol, 7.5 ml concentrated HCl and 105 ml distilled water. Supernatants were combined and adjusted to pH 5.2 followed by the addition of 1 ml of 2 M ammonium phosphate buffer (pH 5.2) per 85 ml extract. Two volumes of cold anhydrous ethanol and four volumes of cold anhydrous ether were added, and the mixture allowed to stand for 48 h at room temperature. The resulting precipitate was collected by filtration through Whatman no. 1 paper and redissolved in 1 M acetic acid (3.5 ml$^{-1}$ of original tissue weight). Extracts were dialysed extensively against 0.1 M acetic acid (Spectrapor tubing, molecular weight cut-off 3,500, Spectrum Medical Industries, Los Angeles, CA).

### *Radioimmunoassay for hURO-EGF and hTGF*

Lyophilised extracts were redissolved in 5–10 ml 0.04 M phosphate buffer containing 0.15 M NaCl, 0.1 M EDTA and 0.1% sodium azide at pH 4.5 to optimise peptide dissolution. The assays were not affected by sample pH in the range

4.2–7.2. Extracts were filtered through 0.2 m Acrodisc 13 filters (Gelman Science Ltd, Northampton, UK).

Peptides and specific polyclonal antibodies to hURO-EGF and hTGFα were received as gifts from Dr H. Gregory (ICI plc). The antibody to hTGFα was raised in sheep and the EGF antibody was raised in rabbits.

hURO-EGF (6 kDa) and hTGF (5.6 kDa) were radioiodinated using the Iodogen method. Resultant specific activities were 150–200 Ci $\mu g^{-1}$. Standards, controls and samples were diluted in phosphate buffer pH 4.5. Antibodies were diluted in assay buffer comprising 0.04 M phosphate buffer (pH 7.2) containing 0.15 M NaCl, 0.01 M EDTA, 0.1% sodium azide and 0.5% BSA, to which 4 $\mu l\ ml^{-1}$ normal rabbit or sheep serum was added. The hURO-EGF antibody was used at a dilution of 1 in 20,000 and the hTGFα antibody at 1 in 25,000. Samples, standards and controls (250 μl) were mixed with 250 μl antibody followed by 250 μl $^{125}$I-hTGF (containing 25 pg). Following incubation at 4°C for 72 h, 250 μl of precipitating antibody (donkey anti-rabbit or donkey anti-sheep, IDS, Washington, Tyne and Wear, UK) was added at appropriate dilution and incubation continued for a further 24 h at 4°C. Separation of the resultant precipitate was by centrifugation at 2500 r.p.m. at 4°C for 30 min followed by aspiration of supernatant.

The antibody to hTGFα shows non cross-reaction with hURO-EGF at a 2,000-fold greater concentration and the TGFα RIA is sensitive within the range 25 pg to 12.5 ng. The hURO-EGF antibody shows no cross reaction with hTGFα also at a 2,000-fold greater concentration and the hURO-EGF RIA is sensitive within the range 10 pg to 2.5 ng.

*Reverse phase chromatography*

Normal gastric mucosal extract (500 μl containing 5 mg of acid soluble protein) in phosphate buffer pH 4.5 was applied to a Pep RPC 5/5 HR reverse phase column (Pharmacia Ltd, Milton Keynes, Bucks., UK) previously equilibrated with 0.1% trifluoacetic acid (TFA). The column was then eluted at a flow rate of 1 ml $min^{-1}$ at room temperature with a 0–40% linear gradient of acetonitrile containing 0.1% TFA. One millilitre fractions were collected, lyophilised and redissolved in assay buffer before radioimmunoassay for hURO-EGF and hTGFα.

### Results

Immunoreactive hTGFα was detected in all of the tissue extracts examined (Figure 1); individual results on each sample are available from the authors on request. Although tissues were largely derived from only three adults, and absolute levels varied between individuals, the distribution of hTGFα throughout the gastrointestinal tract mucosa appeared similar. hTGFα levels declined significantly from the gastric mucosa (mean 2,232 pg hTGFα per gram wet weight of tissue) to the duodenal mucosa (396 pg $g^{-1}$) and gradually rose again through the ileum (1,290 pg $g^{-1}$) and ascending colon (2,173 pg $g^{-1}$) to decrease again through to the sigmoid colon mucosa (530 pg $g^{-1}$).

EGF immunoreactivity was detected in all but two of the tissue extracts, but levels (0–216 pg hURO-EGF per gram wet weight of mucosa) were much lower than hTGFα levels in the same tissue extracts. There was no apparent pattern in the distribution of hURO-EGF along the gastrointestinal tract mucosa.

To verify the nature of the observed immunoreactivity, reverse phase chromatography was carried out on samples of gastric mucosal extracts (Figure 2). hTGFα and hURO-EGF immunoreactivity were found to coelute exactly with hTGFα and hURO-EGF standards previously applied to the column. hTGFα eluted at 25% acetonitrile (peak retention 36 ml) and hURO-EGF at 30% acetonitrile (peak retention 42 ml).

Gastrointestinal mucosal extracts from a 7-year-old female contained higher levels of hTGFα than corresponding adult tissue extracts, with the exception of that derived from the

**Figure 1** Human transforming growth factor α (□) and human urogastrone (■) epidermal growth factor mean values (ng per g tissue wet weight) in the mucosa from normal human adult stomach, duodenum, jejunum, ileum, ascending colon, transverse colon, descending colon and sigmoid colon. Measured by specific radioimmunoassays.

**Figure 2** Profile of extract of normal human gastric mucosa obtained by reversed phase chromotography on FPLC using an acetonitrile gradient (■) showing separation of peptide peaks and coinciding positions of elution of pure hTGF (●) and URO-ERG (○) with the peaks obtained in the biological extract.

stomach (Figure 3), and the distribution of hTGFα was also markedly different. The level of hTGFα was also markedly different. The level of hTGFα in the gastric mucosa (2,200 pg per gram wet weight of tissue) was similar to the average adult level, but this level was maintained throughout the duodenum and jejunem, rising to 5,042 pg $g^{-1}$ in the ileum. hTGFα levels did not decline in the large bowel with 5,119 pg $g^{-1}$ present in the sigmoid colon. hURO-EGF was not detected in the mucosal extracts from this individual.

Extracts of submucosal tissues from the same regions of the gastrointestinal tract of the 7-year-old were also examined, but neither hURO-EGF nor hTGFα were detected in these samples. The apparent absence of hURO-EGF in these submucosal samples was supported by routine immunocytochemical analysis (results not shown), which also failed to detect hURO-EGF in duodenal Brunner's glands at

**Figure 3** Levels of hTGFα seen in different regions of gastrointestinal mucosa from a 7-year-old female. hURO-EGF levels were undetectable.

this age. The avidin-biotin method employed the same antibody used for hURO-EGF radioimmunoassay, and adult control tissue showed positive staining for EGF in Brunner's glands of the duodenum (data on file).

### Discussion

The presence of hURO-EGF in human submandibular glands, saliva, stomach, gastric juice and Brunner's glands, together with the finding that the hURO-EGF receptor is expressed throughout the gastrointestinal mucosa, has led to the proposal that this peptide plays a role in normal gastrointestinal epiithelial maintenance. Recent studies in the rabbit suggest that hURO-EGF may play an important role in post-natal hepatic growth and maturation (Opleta *et al.*, 1987). The present study demonstrated that hTGFα, which also binds to and activates the hURO-EGF receptor kinase, is present throughout the normal gastrointestinal mucosa in significantly higher quantities than hURO-EGF. This finding suggests that this peptide, which until recently was thought to be tumour and embryo specific may be also involved in the control of normal cell renewal in the epithelial lining of the gut.

The possibility that these peptides are not produced by the gastrointestinal mucosa but are sequestered by them cannot be discounted. Indeed, the low levels of hURO-EGF detected in most of the adult tissues may represent the peptide contained within platelets present in the mucosal samples prior to extraction. Previous work in our laboratory has shown that up to 11 ng of hURO-EGF per ml of serum can be released from platelets following the storage of whole blood from normal individuals. We have not, however, detected hTGFα in the platelets or serum of normal individuals ($n = 6$) or of patients suffering from gastrointestinal cancer ($n = 12$) (unpublished observations).

The presence of hTGFα in the stomach and duodenum, implies the simultaneous production of two distinct molecules with affinities for the same receptor. EGF in intracellular granules positive by the immunoperoxidase technique have been described (Elder *et al.*, 1986), but TGFα specific localisation is not kown. The reasons for the presence of these highly similar peptides in the same tissue remain obscure. However, Derynck (1986) suggest that there are subtle differences in cellular responses to the binding of EGF and TGFα in specific *in vitro* and *in vivo* experimental models. For example, TGFα is reported to elicit a greater effect in inducing the formation of epidermal cell colonies in soft agar than does EGF; TGFα appears more effective than EGF in the induction of cell ruffling (Myrdal, 1985) and is more potent in stimulating osteoclast precursor cells (Ibbotson *et al.*, 1986). TGFα has also been shown to induce neovascularisation in hamster cheek pouches at much lower concentrations than EGF (Schreiber *et al.*, 1986), and was much more effective than EGF in terms of producing a maximum increase in blood flow in an experimental model (Gan *et al.*, 1987). Moreover TGFα appears to have the ability to regulate vascular reactivity without desensitisation as is seen with EGF and Gan *et al.* have pointed that this may have a potential role in the progression of tumours secreting TGFα.

Coffey *et al.* (1987) have recently reported that the addition of EGF or TGFα to primary cultures of neonatal human keratinocycles induces TGFα gene expression. They propose that this possible autoregulation of cell proliferation could be responsible for amplification of the growth factor response. If this is indeed the case, the release of EGF in gut epithelium, following a specific stimulus, could result in the autocrine secretion of TGFα and the possibility of subsequent cell proliferation.

The profile of hTGFα levels along the adult gastrointestinal tract mucosa shows lower levels in the duodenum and jejunum. Since hURO-EGF has been localised to the submucosal Brunner's glands, a reciprocal inverse relationship may exist between the two peptides in these areas of rapid cell renewal. Distally, hTGFα levels rise first and then decline again along the colon. It is interesting to note that lower levels of hTGFα and very little hURO-EGF are present in the descending and sigmoid colon, the most common sites of gastrointestinal tumour formation. In contrast, hTGFα levels detected in the extracts of colonic mucosa derived from the juvenile did not decline and in the sigmoid colon were approximately 10 times higher than in the corresponding adult tissue extracts. Colonic cancers are virtually unknown in children. However, only one juvenile has been studied and the distribution described requires confirmation by further reports.

Little is known in the human species about the regulation of hURO-EGF or hTGFα synthesis. Studies in the mouse suggest that EGF concentration in the submandibular gland is influenced by androgens, thyroid hormones, progesterone and oestrogen, probably by means of alteration of the synthesis of prepro-EGF (Gresik *et al.*, 1981; Walker *et al.*, 1981; Bullock *et al.*, 1975; Kurachi & Oka, 1986; Grubits *et al.*, 1986). Further studies are now required to determine if the gut is a target organ for these well established regulatory hormones as regards EGF and TGFα synthesis. The area of origins, actions, physiological roles and relationship between production of EGF or TGFα to cancer induction and metastasis has recently been reviewed (Burgess, 1989), but evidence is mounting that these peptides may be of much more physiological than pathological importance.

We should like to thank Mrs Louise Ellis of the North Staffordshire Medical Institute for typing the manuscript and the Department of Medical Illustration, North Staffordshire Royal Infirmary for the charts and photographs.

### References

ANZANO, M.A., ROBERTS, A.B., MEYERS, C.A. & 4 others (1982). Synergistic interaction of two classes of transforming growth factors from murine sarcoma cells. *Cancer Res.*, **42.** 4776.

BULLOCK, L.P., BARTHE, P.L., MOWSZOWICZ, I. & 2 others (1975). The effects of progestins on submandibular gland epidermal growth factor: demonostration of androgenic, synandrogenic and antiandrogenic actions. *Endocrinology*, **97,** 189.

BURGESS, A.W. (1989). Epidermal growth factor and transforming growth factor α. *Br. Med. Bull.*, **45,** 401.

COFFEY, R.J., DERYNCK, R., WILCOX, J.N. & 4 others (1987). *Nature*, **328,** 817 (letter).

DERNYCK, R. (1986). Transforming growth factor α: structure and biological activities. *J. Cell Biochem.*, **32,** 293.

ELDER, J.B., WILLIAMS, G., LACEY, E. & 1 other (1978). Cellular localisation of human urogastrone/epidermal growth factor. *Nature*, **271,** 466.

ELDER, J.B., HILEY, C. & GREGORY, H. (1986). Epidermal growth factor–Urogastrone (hEGF-URO); Localisation in the gut. *Can. J. Physiol Pharm.*, **5,** 148 (abstract).

GAN, B.S., HOLLENBURG, M.D., MACCANNELL, K.L. & 3 others (1987). Distinct vascular actions of epidermal growth factor Urogastrone and transforming growth factor α. *J. Pharmacol. Exp. Ther.*, **242,** 331.

GOUSTIN, A.S., LEOF, E.B., SHIPLEY, G.D. & 1 other (1986). Growth factors and cancer (review). *Cancer Res.* **46,** 1015.

GREGORY, H. (1975). Isolation and structure of urogastrone and its relationship to epidermal growth factor. *Nature*, **257,** 325.

GREGORY, H., WALSH, S. & HOPKIN, C.R. (1979). The indentification of Urogastrone in serum, saliva and gastric juice. *Gastroenterology*, **77,** 313.

GRESIK, E.W., SCHENKEIN, I., VAN DER NOLN, H. & 1 other (1981). Hormone regulation of epidermal growth factor and protease in the submandibular gland of adult mouse. *Endocrinology*, **109,** 924.

GRUBITS, R.M., SHAW, P.A., GRESIK, E.W., ONETTI-MUDA, A. & BARKA, Y. (1986). Epidermal growth factor gene expression is regulated differently in mouse kidney and submandibular gland. *Endocrinology*, **119,** 1382.

HIRATA, Y. & ORTH, D.N. (1979). Epidermal growth factor (urogastrone) in human fluids; size heterogenity. *J. Clin. Endocrinol. Metab.*, **48,** 673.

IBBOTSON, K.J., HARROD, J., GOWEN, M. & 5 others (1986). Human recombinant transforming factor α stimulates bone resorption and inhibits formation *in vitro*. *Pro. Natl Acad. Sci. USA*, **83,** 2228.

KIM, M.K., WARREN, T.C. & KIMBALL, E.S. (1985). Purification and characterisation of a low molecular weight transforming growth factor from the urine of melanoma patients. *J. Biol. Chem.*, **260,** 9237.

KURACHI, H. & OKA, T. (1986). Regulation of the level of epidermal growth factor by oestrogen in the submandibular gland of female mice. *J. Endocrinol.*, **109,** 221.

MARQUARDT, H., HUNKAPILLAR, M.W., HOOD, L.E. & 4 others (1983). Transforming growth factors produced by retrovirus transformed rodent fibroblasts and human melanoma cells: amino acid sequence homology with epidermal growth factor. *Proc. Natl Acad. Sci. USA*, **80,** 4684.

MYRDAL, S. (1985). Differences in early cellular responses to epidermal growth factor and transforming growth factor type-α. *J. Cell Biol.*, **101,** 244A.

OKA, Y. & ORTH, D.N. (1983). Plasma epidermal growth factor/urogastrone is associated with blood platelets. *J. Clin. Invest.*, **72,** 249.

OKAMATO, S. & OKA, T. (1984). Evidence for physiological function of epidermal growth factor: pregestational sialodenectomy of mice, decreases milk production and increases offspring mortality during the lactation period. *Proc. Natl Acad. Sci. USA*, **81,** 6059.

OPLETA, K., O'LOUGHLIN, E.V., SHAFFER, E.A. & 3 others (1987). Effect of epidermal growth factor on growth and post natal development of the rabbit liver. *Am. J. Physiol.*, **253** (Gastrointest. Liver Physiol. **1**), G622.

ROBERTS. A.B., LAMB, L.C., NEWTON, D.L. & 3 others (1980). Transforming growth factors - Isolation of polypeptides from virally and chemically transformed cells by acid/ethanol extraction. *Proc. Natl Acad. Sci. USA*, **77,** 3494.

SCHRIEBER, A.B., WINKLER, M.E. & DERYNCK, R. (1986). Transforming growth factor α: a more potent angiogenic factor than epidermal growth factor. *Science*, **232,** 1250.

SMITH, J.M., SPORN, M.B., ROBERTS, A.B. & 3 others (1985). Human transforming growth factor causes precocious eyelid opening in newborn mice. *Nature*, **315,** 515.

SPORN, M.B. & ROBERTS, A.B. (1985). Autocrine growth factors and cancer. *Nature*, **313,** 745.

SPORN, M.B., & ROBERTS, A.B. (1986). Peptide growth factors and inflammation, tissue repair and cancer. *J. Clin. Invest.*, **78,** 329.

ULLRICH, A., COUSSENS, L., HAYFLICK, J.S. & 12 others (1984). Human epidermal growth factor receptor cDNA sequence and aberrant expression of the amplified gene in A431 epidermoid carcinoma cells. *Nature*, **309,** 418.

WALKER, P., WEICHSEL, M.E.Jr., HOATH, S.B. & 2 others (1981). Effects of thyroxin, testosterone and corticosterone on nerve growth factor (NGF) and epidermal growth factor (EGF) concentration in adult female mouse submandibular gland: dissociation of NGF and EGF responses. *Endocrinology*, **109,** 582.

YEH, Y.C., TSAI, J.F., CHUANG, L.Y. & 4 others (1987). Elevation of transforming growth factor α and its relationship to the epidermal growth factor and, α-fetoprotein levels in patients with hepatocellular carcinoma. *Cancer Res.*, **47,** 896.